# Mature autologous dendritic cell vaccines in advanced non-small cell lung cancer: a phase I pilot study

**DOI:** 10.1186/1756-9966-30-65

**Published:** 2011-06-17

**Authors:** Maurício W Perroud, Helen N Honma, Aristóteles S Barbeiro, Simone CO Gilli, Maria T Almeida, José Vassallo, Sara TO Saad, Lair Zambon

**Affiliations:** 1 Department of Internal Medicine, Faculty of Medical Sciences, State University of Campinas, Campinas, Brazil; 2 Hemocentro, State University of Campinas, Campinas, Brazil; 3 Laboratory of Investigative and Molecular Pathology-CIPED, Faculty of Medical Sciences, UNICAMP - Campinas, São Paulo, Brazil

## Abstract

**Background:**

Overall therapeutic outcomes of advanced non-small-cell lung cancer (NSCLC) are poor. The dendritic cell (DC) immunotherapy has been developed as a new strategy for the treatment of lung cancer. The purpose of this study was to evaluate the feasibility, safety and immunologic responses in use in mature, antigen-pulsed autologous DC vaccine in NSCLC patients.

**Methods:**

Five HLA-A2 patients with inoperable stage III or IV NSCLC were selected to receive two doses of 5 × 10^7 ^DC cells administered subcutaneous and intravenously two times at two week intervals. The immunologic response, safety and tolerability to the vaccine were evaluated by the lymphoproliferation assay and clinical and laboratorial evolution, respectively.

**Results:**

The dose of the vaccine has shown to be safe and well tolerated. The lymphoproliferation assay showed an improvement in the specific immune response after the immunization, with a significant response after the second dose (p = 0.005). This response was not long lasting and a tendency to reduction two weeks after the second dose of the vaccine was observed. Two patients had a survival almost twice greater than the expected average and were the only ones that expressed HER-2 and CEA together.

**Conclusion:**

Despite the small sample size, the results on the immune response, safety and tolerability, combined with the results of other studies, are encouraging to the conduction of a large clinical trial with multiples doses in patients with early lung cancer who underwent surgical treatment.

**Trial Registration:**

Current Controlled Trials: ISRCTN45563569

## Background

Lung cancer is the leading cause of cancer-related morbidity and mortality, resulting in more than 1 million deaths per year worldwide[1]. In Brazil, the current estimatives of incidence are 18.37/100.000 and 9.82/100.000 for men and women, respectively[2]. About 70% of patients with lung cancer present locally advanced or metastatic disease at the time of diagnosis, because there is no efficient method to improve the early diagnosis[3] and this fact has a huge impact on treatment outcomes. In spite of the aggressive treatment with surgery, radiation, and chemotherapy, the long-term survival for patients with lung cancer still remains low. Even patients with early stage disease often succumb to lung cancer due to the development of metastases, indicating the need for effective approaches for the systemic therapy of this condition [4].

A variety of novel approaches are now being investigated to improve the outlook for management of this disease. Theories have also been postulated regarding the failure of the immune systems to prevent the growth of tumors. However, despite significant advances in our understanding of the molecular basis of immunology, many obstacles remain in translating this understanding into the clinical practice in the treatment of solid tumors such as lung cancer[1].

Dendritic cells (DCs) are the most potent antigen presenting cells with an ability to prime both a primary and secondary immune response to tumor cells. DCs in tumors might play a stimulating and protective role for effector T lymphocytes, and those DCs that infiltrate tumor tissue could prevent, by co-stimulating molecules and secreting cytokines, tumor-specific lymphocytes from tumor-induced cell death[5].

We believe that tumor vaccines may play an adjuvant role in NSCLC by consolidating the responses to conventional therapy. Then, we decided to conduct this study to evaluate the feasibility, safety, tolerability and immunologic responses in use in mature, antigen-pulsed autologous DC vaccine in a group of non-small cell lung cancer patients (NSCLC).

## Methods

### Patient Characteristics

Patients who met the following eligibility criteria were included: histopathologically confirmed diagnosis of advanced NSCLC (stage IIIB-IV)[6]; aged ≤70 years; performance status ≤2[7]; no prior chemotherapy, surgery, or radiotherapy; no central nervous system metastases and at least one measurable lesion according to the RECIST's criteria[8]; no associated acute disease; HLA-A2 phenotype and expression of WT1 (*Wilms Tumor Protein*), HER-2 (*Human Epidermal Growth Factor Receptor 2*), CEA (*Carcinoembryonic Antigen*) or MAGE1 (*Melanoma Antigen 1*) proteins at the tumor site (tissue). The phenotype HLA-A2 was chosen due the methodology adopted for the incorporation of the antigen to the dendritic cell. The maintenance of organic functions was confirmed by: white blood cells (WBC) ≥3000/mm^3^, neutrophil cells ≥1500/mm^3^, hemoglobin (Hgb) ≥9.0 g/dL, and platelets ≥100,000/mm^3^; bilirubin ≤1.5 mg/dL, aspartate aminotransferase ≤40 IU/L; creatinine clearance >55 mL/minute. The written informed consent was obtained from all patients enrolled in the study. The study was conducted in accordance with the International Conference on Harmonization (ICH) guidelines, applicable regulations and the guidelines governing the clinical study conduct and the ethical principles of the Declaration of Helsinki.

### Trial Design

**Figure 1:**
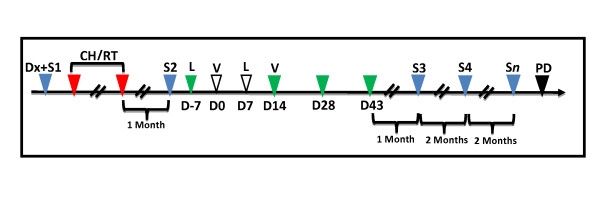
**The steps of the study**. Leukapheresis' day is marked with "L" (D-7 and D7). Immunizations' day is marked with "V" (D0 and D14). Blue triangle - Evaluation step: "Dx+S1" = Diagnosis and 1^st ^Radiologic Staging; "S2" = 2^nd ^Radiologic Staging (1 month after conventional treatment); "S3" = 3^rd ^Radiologic Staging (1 month after vaccine); "S4...S*n" *= Radiologic staging was repeated every 2 months until the progression of the disease ("PD" - black triangle). Red triangle - Conventional treatment (chemo/radiotherapy). Green triangle - Lymphoproliferation test; it was done before immunization on D0 and D14.

The trial was nonrandomized. All selected patients received conventional treatment (chemotherapy with or without radiotherapy). Briefly, the chemotherapy protocols included paclitaxel 175 mg/m^2 ^and cisplatinum 70 mg/m^2 ^on day 1. These cycles were then repeated four times every 21 days. After the forth chemotherapeutical cycle, the patients were submitted to computed tomography (CT) scan of thorax, abdomen and brain to evaluate the tumor response. The progressive disease was an exclusion criterion. Patients who met all criteria for inclusion were eligible to the dendritic cells vaccine as an adjuvant therapy, which was administered after hematological recovery (platelets ≥70,000/mm^3^). The measurable immunologic response and safety to the vaccine were the primary and secondary endpoints. The small sample size could preclude meaningful assessment of therapeutic effects. The clinical tolerability was determined by routine safety laboratories and the clinical events described by the Cancer Therapy Evaluation Program (CTEP), and Common Terminology Criteria for Adverse Events (CTCAEv3)[9]. The steps of the study are showed in figure 1.

### Leukapheresis

Fresenius Com.Tec (Fresenius Kabi - Transfusion Technology, Brazil) was used for all running procedures of the MNC program, at 1500 rpm, and with a P1Y kit. Plasma pump flow rates were adjusted to 50 mL/min. The volume processed ranged between patients and was determined by estimated cell count after 150 mL of processed blood. ACD-A was the anticoagulant used in these studies. The Inlet/ACD Ratio ranged from 10:1 to 16:1. There was no need for replacement, because the total volume of blood taken was less than 15%.

### Microbiologic Monitoring

Microbiological tests were performed at the beginning of the culture, on the fifth day and at the time of vaccine delivery. Samples were incubated for 10 days for the certification of absence of contamination.

### Generation of dendritic cells

After informed consent, the mature dendritic cells of autologous mononuclear cells were isolated by the Ficoll-Hypaque density gradient centrifugation (Amersham, Uppsala, Sweden). Monocytes were then enriched by the Percoll hyperosmotic density gradient centrifugation followed by two hours of adherence to the plate culture. Cells were centrifuged at 500 *g *to separate the different cell populations. Adherent monocytes were cultured for 7 days in 6-well plates at 2 × 10^6 ^cells/mL RMPI medium (Gibco BRL, Paisley, UK) with 1% penicillin/streptomycin, 2 mM L-glutamine, 10% of autologous, 50 ng/mL GM-CSF and 30 ng/mL IL-4 (Peprotech, NJ, USA ). On day 7, the immature DCs were then induced to differentiate into mature DCs by culturing for 48 hours with 30 ng/mL interferon gamma (IFN-γ).

According to the previous expression detected by immunohistochemistry, the HLA-A2 restricted to WT1 peptide (RMFPNAPYL), CEA peptide (YLSGANLNL), MAGE-1 peptide (KVAELVHFL), and HER-2 peptide (KIFGSLAFL) were pulsed to the DC culture (day 9) at the concentration of 25 ug/mL and incubated for 24 hours to the vaccine administration.

### Flow cytometry

DC were harvested on day 7 and washed with PBS. Fluorescent conjugated monoclonal antibodies targeted against the following antigens were used for phenotypic analysis: CD14 (PerCp), CD80 (Pe), CD83 (APC), CD86 (Fitc), HLA-A (Fitc), HLA-DR (Pe-Cy7), CD11c (Pe), CD40 (PerCp-Cy5.5), CCR5 (Pe), CCR7 (Fitc), IL-10 (Pe) and IL-12p70 (Fitc) (Caltag, Burlingame, CA, USA). Antibodies targeted against CD3 (Pe), CD8 (PE-Cy7), CD4 (PerCp) and IFNγ (Fitc) were used for phenotypic analysis of lymphocyte after the lymphoproliferation assay. Isotype-matched antibodies were used as controls (Caltag, Burlingame, CA, USA). The labeling was carried out at room temperature for 30 minutes in PBS. For the intracellular labeling (IL-10 and IL-12p70), cells were permeabilized and fixed using the Fix-Cells Permeabilization Kit (Caltag, Burlingame, CA, USA). After labeling, cells were washed twice in PBS and analyzed by a FACSArea cytometry using the CELL QUEST PRO software application. The DC and lymphocyte populations were gated based on their forward-scatter and side-scatter profile (large or small granular cell population, respectively). The results are expressed as percentage of positive cells and for IL-12 and IL-10 expression, the mean fluorescence intensity was also observed.

### CFSE Labeling

PBMCs (1 × 10^7^) were incubated at 37°C for 15 min in 1 mL of PBS containing CFSE (Molecular Probes Europe, Leiden, The Netherlands) at 0.6 *μ*M, a concentration which was determined in preparatory experiments as useful. After one washing step in PBS containing 1% FCS, the cells were re-suspended at a density of 1 × 10^6 ^cells/mL and used to perform the lymphoproliferation assay. After 6 days of incubation, the CFSE-labeled cells were washed once in PBS and then either immediately fixed in PBS containing 4% formaldehyde, and subjected to analysis by a FACSArea and CellQuest software (BD, Mountain View, CA, USA). The CFSE-fluorescence was plotted against forward scatter. The retained bright CFSE staining consistent with no proliferative response and the lost CFSE-fluorescence indicated an induced proliferation. The reduced level of CFSE staining in the stimulated lymphocyte in relation to the unstimulated was used to calculate a proliferation index.

### Immunization Protocol

A prime vaccine and a single boost were given fifteen days apart. For each dose of vaccine, two aliquots were prepared in separated syringes with saline solution (500 μl/dose) containing 5 × 10^7 ^cells. First, a dose was subcutaneously administered in the arm and after 1 hour the second dose was given intravenously in the other arm. After the second dose, the patient remained under observation for 1 hour for evaluation of immediate unexpected adverse events.

### Clinical Evaluation

The follow-up included routine history and physical exam, chest x-ray and computed tomography scans at regular intervals post immunization or as directed by signs or symptoms of tumor recurrence.

### Immunologic Assessment

#### A. Phenotypic characterization of immune cells from patients' peripheral blood

The cellular composition of the immune system, before and after vaccination with the dendritic cells, was assessed from peripheral blood samples using flow cytometry. The day of immunization was considered as "Day 0". The peripheral blood samples were collected one week before vaccination ("Day -7"), two weeks after the first dose of vaccine ("Day 14"), two weeks after the second dose of vaccine ("Day 28") and one month ("Day 43") after the end of the vaccination protocol.

Surface antigens labeled with specific fluorochromes for T lymphocytes (CD4 and CD8), NK cells (CD56), B lymphocytes (CD19) and mature dendritic cells (CD86, CD80, CD83, CD40 and HLA-DR) were used for immunophenotyping of the patients' blood cells.

Approximately 2 × 10^5^cells per test were treated with a lysis solution for the red blood cells, centrifuged at 300 *g *for 5 minutes, rinsed with PBS and re-suspended in 100 μl of cytometry buffer (PBS with 0.5% bovine serum albumin and 0.02% sodium azide). Subsequently, these cells were incubated in the dark for 30 minutes at 4°C with monoclonal antibodies labeled with the specific fluorochromes described above. Then the samples were washed twice with flow cytometry buffer, fixed with paraformaldehyde and analyzed by a flow cytometer (FACSCalibur - Becton Dicknson).

#### B. Analysis of the specific immune response in vitro by flow cytometry

The lymphoproliferation test was used to assess the ability of dendritic cells to stimulate specific lymphocytes *in vivo*.

#### C. Collection of T lymphocytes

The peripheral blood samples collected at the times describes above were enriched with T lymphocytes (CD3^+^) by negative immune selection with immunomagnetic beads specific for NK cells (CD56^+^), B lymphocytes (CD19^+^) and monocytes (CD14^+^).

The cells collected before vaccination were centrifuged at 600 *g *during 10 minutes and the cell pellet was washed twice with PBS, re-suspended in RPMI with 1% human AB serum and 10% dimethyl sulfoxide and then frozen to -90° C at a controlled rate of 1° C/minute until the time of the first test (two weeks after the first dose of the vaccine).

#### D. Lymphoproliferation assay

The T cells (1 × 10^6^cels/mL) were re-suspended in 1 mL of PBS containing 0.25 μM of CFSE (Molecular Probes, The Netherlands) and incubated for 15 minutes at 37°C. After this incubation period, the cells were washed twice with RPMI 1640 supplemented with 1% human AB serum cold by centrifugation at 600 *g *for 10 minutes and incubated in ice for 5 minutes.

After this period, the cells were again centrifuged at 600 *g *for 10 minutes and re-suspended in the same medium supplemented with 25 ng/mL of IL-7. These lymphocytes were cultivated in 24-well plates (1 × 10^5 ^cells/well) with 25 μg/mL of each tumor peptide defined for each patient, separately. This culture was incubated for 4 days at 37°C in 5% CO_2_.

The percentage of proliferation was calculated using the number of cells with CFSE labeling using the following formula: [(Number of CFSE-labeled cells in the test group - Number of CFSE-labeled cells in the control group)/Number of CFSE-labeled cells in the control] × 100. As for the control, the same test was performed using unstimulated lymphocytes labeled with CFSE. All tests had been carried out in triplicate.

The results of the lymphoproliferation were compared using Wilcoxon signed ranks test.

## Results

### Patient Characteristics

Between June/2006 and August/2008, 48 patients were evaluated. Only five patients met all criteria for inclusion in the study. The median age was 60 years and 3 of 5 patients were males. The histologic subtypes were as follows: adenocarcinoma (2), invasive mucinous adenocarcinoma (former bronchioloalveolar) (1), squamous cell carcinoma (1) and adeno/squamous cell carcinoma (1). Four patients were stage IIIB and one was stage IV at the time of the diagnosis. The patients' characteristics are summarized in Table 1.

**Table 1:** Patient characteristics

Patient ID	Sex	Age	Histology	Stage at enrollment	ECOG*	Expression	Therapy Sequence	Time between the treatment modalities (days)	Response to the conventional treatment (RECIST)	Time to progression from Chemotherapy (days)	Time to progression from Immunotherapy (days)	Survival from Diagnosis (days)	Survival from Immunotherapy (days)
**1**	M	61	Sq/Ad	IIIB (T4,N2)	1	HER-2 (grade 3) MAGE1 (grade 5)	CT - IT	77	Partial Response	138	47	258	84
**2**	M	66	Ad	IIIB (T2,N3)	2	WT1 (grade 4) CEA (grade 6)	CT - IT - XRT	38; 3	Stable disease	112	60	358	198
**3**	M	59	Ad	IIIB (T4,N2)	1	CEA (grade 7)	CT - XRT - IT	30; 52	Stable disease	231	82	276	112
**4**	F	63	IMA	IV (T4,N2,M1)^#^	2	WT1 (grade 2) CEA (grade 7) HER-2 (grade 1)	CT - IT - CT	45; 56	Stable disease	64	1	329	82
**5**	F	50	Sq	IIIB (T4,N2)	1	CEA (grade 3) HER-2 (grade 2)	CT - XRT - IT	51; 56	Partial Response	200	22	560	277

Sq, squamous cell carcinoma; Ad, adenocarcinoma; IMA, invasive mucinous adenocarcinoma.

*ECOG: Eastern Cooperative Oncology Group performance status.

#T4Ipsi Nod, N2,M1aCont Nod

### Safety

During the chemo and radiotherapy, no adverse events grade >2 were reported. No reaction was observed during or after the leukapheresis. No local reaction was observed at the vaccine site of application. One patient presented systemic reactions after the immunotherapy. This patient developed fatigue (grade 2) and chills five days following the first dose of the vaccine and was hospitalized on the 7^th ^day because the laboratorial analyses showed leukopenia (1,500/mm^3^; grade 3), granulocytopenia (900/mm^3^; grade 3), lymphopenia (495/mm3; grade 3); thrombocytopenia (88,000/mm3; grade 1); anemia (hemoglobin 8,5 g/dL; grade 2) and hyponatremia (126 mEq/L; grade 3). The serology to the Human Immunodeficiency Virus (HIV), mononucleosis, cytomegalovirus, Epstein Barr, *Mycoplasma pneumoniae *and dengue were negatives, as well as the bacterial cultures. Cephepime was prescribed empirically. No colony-stimulating factor was used and the patient recovered from blood changes, spontaneously, after five days, except by the anemia. The hyponatremia was treated with sodium replacement and became normal after one week.

### Immunologic responses to Vaccines

The lymphoproliferation assay showed an improvement in the specific immune response after the immunization. This response was not long lasting and a tendency to reduction 2 weeks after the second dose of the vaccine was observed.

**Figure 2:**
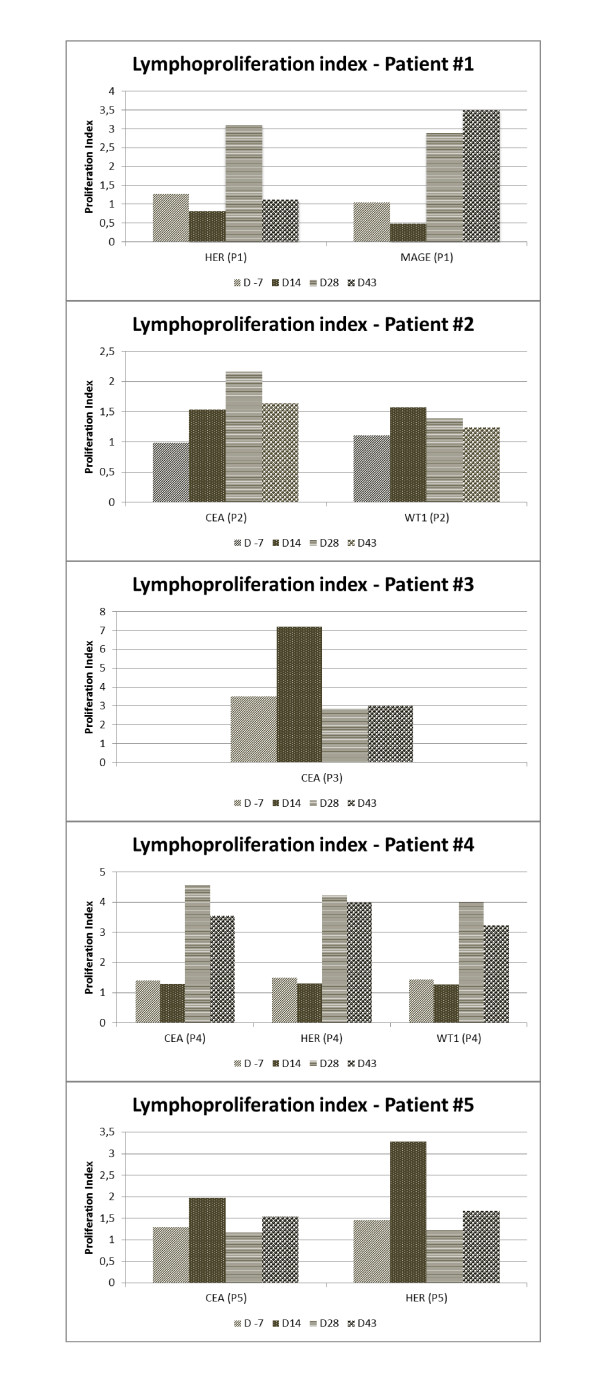
**Immunological response**. Lymphoproliferation index: "D -7" (1 week before 1^st ^dose); "D 14" (2 weeks after 1^st ^dose); "D 28" (2 weeks after 2^nd ^dose); "D 43" (4 weeks after 2^nd ^dose); HER, human epidermal growth factor receptor; MAGE, melanoma antigen; CEA, carcinoembryonic antigen; WT1, Wilms tumor protein; P1, patient 1; P2, patient 2; P3, patient 3; P4, patient 4; P5, patient 5.

Patterns of reactivity ranged between individuals (Figure 2). Two patients (#3 and #5) expressed a noteworthy result at the lymphoproliferation tests at one time point after the first dose. Patients #1 and #4 presented a visibly boosted response temporally related to the second dose. Patient #2 showed a mixed response with a strongest response after the first dose to WT1 and a boosted response to CEA.

**Figure 3:**
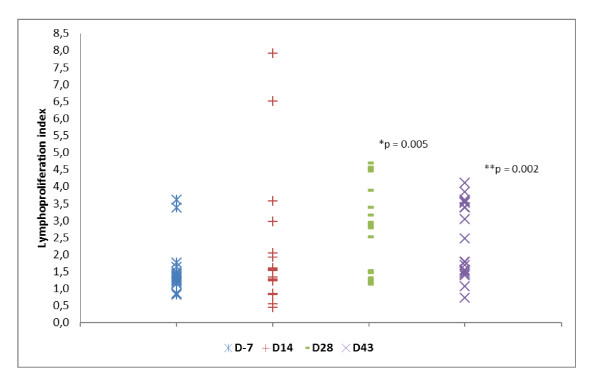
**Immunological response**. Lymphoproliferation's results from all patients and all antigens were compared using Wilcoxon signed ranks test. "D -7" (Median = 1.33; Min = 0.81; Max = 3.59); "D 14" (Median = 1.42; Min = 0.44; Max = 7.90); "D 28" (Median = 2.86; Min = 1.13; Max = 4.68); "D 43" (Median 2.13; Min = 0.72; Max = 4.10). The difference was significant between "D -7" and "D 28" (*p = 0.005) and "D-7" and "D-43" (**p = 0.002).

All the results of the lymphoproliferation assay - all patients and all antigens - are showed in Figure 3. These results were compared using Wilcoxon signed ranks test. The difference between "D-7" and "D 14" was not significant (*p *= 0.135). However, the difference was significant between "D -7" and "D 28" (*p *= 0.005) and between "D -7" and "D 43" (*p *= 0.002).

### Clinical outcomes

The clinical follow-up was available for all individuals for a minimum of 8.5 months from the diagnosis and almost 3 months from de second dose of immunotherapy. Data are presented in Table 1. Two individuals had partial response to the conventional therapy, while three had a stable disease. All of them received chemotherapy and those three were submitted to radiotherapy as well. Patient #2 underwent immunotherapy previous to the radiotherapy. From the last dose of the vaccine, the time to the disease progression and survival ranged between 1 to 82 and 82 to 277 days, respectively. One day after immunotherapy, the Patient # 4 presented worsening of the cough accompanied by progressive dyspnea. The follow up showed progressive disease on the radiologic exams.

## Discussion

Despite the developments on chemo and radiotherapy, the 5 year survival rate improved only 3% (13 to 16.2%) between 1975 and 2002[10]. This fact occurs mainly because there is not an efficient screening method for the early diagnosis and it also shows that new therapeutic modalities are necessary.

Based on the antigen specificity of the immune system and the safety profile of cancer vaccines, the effective immunotherapy would be an ideal adjuvant, following initial clinical responses to definitive therapy[11]. The antigen-presenting cells, like dendritic cells, play an important role in the induction of an immune response, and an imbalance in the proportion of macrophages, immature and mature dendritic cells within the tumor could significantly affect the immune response to cancer [4].

Even though there have been numerous clinical trials for various types of cancer, there are few DC vaccines trials in patients with lung cancer, and many aspects related to the immunotherapy - like maximum dose, administration schema, response and safety - are unknown.

Our study was done with two aliquots of 5 × 10^7 ^cells for each dose. This dose is similar to that of other studies that used doses ranging between 8.2 and 10 × 10^7 ^cells[11-13]. Another trial demonstrated that a dose of 1.2 × 10^7 ^cells did not reach a truly maximum tolerated dose[14]. Given that there is no clear consensus about whether or not the route of immunotherapy influences on the efficacy of the vaccine, we chose to apply it by a subcutaneous and intradermal route.

In addition to the high level dose, the vaccine was well-tolerated as noted in many studies[11-15], even in a study in Hepatitis C Virus (HCV) infected individuals[16]. We observed no local reaction, but one patient presented fatigue, chills, pancytopenia and hyponatremia five days after the first dose of the vaccine. Usually, the reactions after immunotherapy occur within 24-48 hours after the infusion[12,17]. Therefore, we hypothesize that the patient developed an infection, but it cannot be proved because the bacterial cultures and viral tests were negatives.

Three patients had a longer time survival than expect for their TNM stage. Two of these (patients #4 and #5) had a survival almost twice greater than the expected average and they were the only ones that expressed HER-2 and CEA together. Although the small sample size precludes the meaningful assessment of the therapeutic effects and any results may be due to chance, we cannot exclude that these clinical outcomes may indicate some therapeutic efficacy. Many variables related to the host and the vaccine may be important to reach therapeutic efficacy. The immunologic resistance of a tumor to immune effector cells at the local level remains a potential limitation to the vaccine efficacy, and the choice of antigens is also relevant to the therapeutic efficacy and potentially to the immunologic responses to vaccines[12]. Furthermore, the characteristics of the tumor antigen may change and it can become unresponsive to the initial tumor-antigen targeted therapy as tumors grow during conventional therapy[14,15]. We decided to produce a multivalent vaccine according to each patient tumor's antigen expression, observed by immunohistochemistry, to avoid this phenomenon and improve the results of immunotherapy by inducing a broad repertoire of antigen-specific T cells[15]. Indeed, the profile of antigens with better therapeutic responses has not yet been determined.

The patterns of reactivity ranged between individuals (Figure 2). Two patients expressed a significant immunologic reaction after the first dose; another two presented a boosted response after the second dose and one showed a mixed response. The lymphoproliferation assay showed an improvement in the specific immune response after the immunization (Figure 3). However, this response was not long lasting and a tendency to reduction 2 weeks after the second dose of the vaccine was observed. This finding is consistent with other studies that showed a booster response to subsequent immunization[11,12]. The trend to return to baseline after an increase of reactive T cells might be viewed as a transient response[11], associated to the immunosuppressive environment within a tumor mass. It turns the vaccination protocol into a tiresome activity given that multiples doses may be required to reach clinical efficacy.

## Conclusion

Despite the small sample size, the results on the immune response and safety, combined with the results from other studies, are encouraging to the conduction of a clinical trial with multiples doses in patients with early lung cancer who underwent surgical treatment. The DC vaccine could be a hopeful adjuvant therapeutic modality for this group of patients because they do not present a gap to antigenic changes or a bulky disease.

## List of Abbreviations

DC: dendritic cell; NSCLC: non-small-cell lung cancer; WT1: Wilms Tumor Protein; HER-2: Human Epidermal Growth Factor Receptor 2; CEA: Carcinoembryonic Antigen; MAGE1: Melanoma Antigen 1.

## Competing interests

The authors declare that they have no competing interests.

## Authors' contributions

STS and LZ conceived the design of the study, participated in data analysis and were in charge of its coordination. JV and HNH processed the tumor tissue and performed the immunohistochemistry. ASB and MWP cared for the patients during the conventional treatment. MWP and SCOG cared for the patients during the immunotherapy, participated in data analysis, performed data interpretation and drafted the manuscript. MTA conducted the laboratory procedures to produce the DC vaccine, supported by SCOG. All authors read and approved the final manuscript.
